# B4GALNT2 Gene Promotes Proliferation, and Invasiveness and Migration Abilities of Model Triple Negative Breast Cancer (TNBC) Cells by Interacting With HLA-B Protein

**DOI:** 10.3389/fonc.2021.722828

**Published:** 2021-09-13

**Authors:** Pu Yu, Lili Zhu, Kang Cui, Yabing Du, Chaojie Zhang, Wang Ma, Jia Guo

**Affiliations:** ^1^Department of Oncology, The First Affiliated Hospital of Zhengzhou University, Zhengzhou, China; ^2^Nephrology Research Center, The First Affiliated Hospital of Zhengzhou University, Zhengzhou, China

**Keywords:** triple negative breast cancer, invasiveness, migration, B4GALNT2, HLA-B protein

## Abstract

B4GALNT2 gene encodes the enzyme β1,4-N-acetylgalactosaminyltransferase 2 that biosynthesizes the histo-blood group antigen Sd^a^, which is expressed on the surface of erythrocytes and in body secretions. Analysis of The Cancer Genome Atlas (TCGA) database revealed that this gene was highly expressed in breast cancer tissues in comparison with adjacent healthy ones. *In-vitro* lentivirus-assisted B4GALNT2 gene knockdown experiments in model triple negative breast cancer (TNBC) cell lines (HCC1937 and MDA-MB-231) showed inhibition in cell proliferation, decrease in cell viability, promotion of cell apoptosis and inhibitions in cell migration and invasiveness abilities in comparison with empty lentivirus transfectant controls. Also, in cell cycle tests, the number of cells in the G1 phase increased, in the S phase decreased and did not change in the G2/M phase (indicative of the presence of a block in the G1 phase). *In-vivo* tumor formation experiments in mice revealed that knockdown of the B4GALNT2 gene in MDA-MB-231 cells inhibited their proliferation. Using co-immunoprecipitation (Co-IP) mass spectroscopy-assisted analysis, it was found that HLA-B protein [a product of the human leukocyte antigen (HLA) class I gene] interacts with B4GALNT2 protein. *In-vitro* overexpression of HLA-B in B4GALNT2-knocked down MDA-MB-231 cell lines significantly recovered the cell proliferation, viability and migration ability of B4GALNT2 gene. These indicate that HLA-B is one of the interaction proteins in the downstream pathway of the B4GALNT2 gene.

## Introduction

Approximately 1 in 8 women is diagnosed with breast cancer during their lifetime (1). Breast cancer accounts for the highest new incidence (24.2%) and mortality (15.0%) for cancer cases diagnosed in women globally (2); it also shares the first place with lung cancer (each 11.6%) for the total (men and women) new incidence of cancer cases, with a mortality (6.6%) ranked after lung, colorectum, stomach and liver cancers (2). For nearly two decades, the global new incidence of breast cancer has steadily increased, from 1.15 million (in 2002) (3) to 1.38 million (in 2008) (4), 1.68 million (in 2012) (5), and 2.09 million (in 2018) (2); the predicted number for 2050 is approximately 3.2 million (6).

Established risk factors/markers of breast cancer could be classified within hereditary, life events, radiation, medication and diet and nutrition categories (7). (i) Breast cancer family history among first degree-relatives increases the risk almost twice (for one cancer history), and about three- to four-times (for more than one cancer history) (8–10). Also, very high risks are associated with germline mutations in BRCA1, BRCA2 and TP53 genes that account for 2-5% of cases (7, 11). (ii) Life events that increase lifetime exposure to oestrogen and progesterone increase the risk, including early menarche, late-onset natural menopause, not bearing children and first pregnancy over the age of 30 (12, 13). Also, the risk doubles each decade until menopause, when the rate of increase slows down dramatically (12). (iii) Breast cancer risk increases upon ionizing radiation exposure from medical treatment (e.g., X-ray), especially during puberty (14, 15). (iv) Alcohol consumption and a marker like adult attained height are associated with increased risks (7). Body fatness and adult weight gain (for postmenopausal breast cancer) and a marker like greater weight at birth (for premenopausal breast cancer) are also associated with higher risks (7). (v) Hormone therapy that involves oestrogen (with or without progesterone) increases the risk (16).

Systemic therapy and locoregional (including surgery and radiation therapy) approaches are adopted for management of breast cancer. The systemic therapy approach includes the following. (i) Endocrine therapy is used for hormone-receptor positive (HR+) tumors. For example, in oestrogen-receptor positive (ER+) disease, 5 years of adjuvant tamoxifen (that binds to and inhibits ER) reduces breast cancer death by about a third (during years 0-14), and recurrence by about a half (during years 0-4) and about a third (during years 5-9) (17). (ii) Chemotherapy is effective for both before surgery (neoadjuvant) and after surgery (adjuvant) circumstances (18). Adjuvant chemotherapy reduced breast cancer mortality by, on average, about one-third (19). (iii) Anti-human epidermal growth factor receptor 2 (anti-HER2) therapy is used for HER2+ disease. For example, for HER2+ patients, treatment with adjuvant trastuzumab for 1 year after chemotherapy is associated with significant improvement in long-term disease-free survival (20). (iv) Bone stabilizing agents may have additional benefits other than those for the bones (21). For example, for postmenopausal women, adjuvant bisphosphonates is associated with a reduced rate of breast cancer recurrence in the bone and an improved breast cancer survival (22). (v) Poly (ADP-ribose) polymerase (PARP) inhibitors could be employed for BRCA mutation carriers. For example, for patients with metastatic breast cancer and a germline BRCA mutation, olaparib monotherapy provides a significant benefit over standard chemotherapy by extending the median progression-free survival and lowering the risk of disease progression or death (23). (vi) Immunotherapy appears to be promising for treatment of breast cancer, especially for the case of triple negative breast cancer (TNBC) tumors [tumors negative for ER, progesterone receptor (PR) and HER2] (24, 25). Examples of immunotherapy with promising outcomes for TNBC patients include immune checkpoint inhibitors, such as atezolizumab and pembrolizumab (26, 27).

Once using an imaging technique an anomaly is found in the breast tissue, for diagnoses, prognosis and treatment purposes, cytological, histological and molecular analysis is applied to biopsies using genetic tests [gene sequencing and gene expression profiling signature (GEP/S) (28–30)] and biochemical markers (31–35). Biochemical markers can be found in tissues (31) and within the circulating blood (36) [e.g., circulating tumor cells (CTCs)]. Established biochemical biomarkers are proteins and comprise of ki6 7 (37), ER [ERα (38) and ERβ (39)], PR (33) and HER2 (40). In addition to these, many investigative biomarkers exist, including proteins (e.g., the glycoprotein CA 15-3 and the enzyme CEA) and nucleic acids. Examples of the latter include, (i) mRNAs [e.g., oestrogen-related receptors (41) (ERRs; including ERRα (42, 43), ERRβ (33) and ERRγ (43))], (ii) miRNAs found in tissues (44, 45) and the circulating blood (46, 47) [e.g., miR-21, miR-125b, miR-145, and miR-155 (44), and miR-195 (46)], and (iii) DNAs [e.g., short cell-free circulating tumor DNA (ctDNA) (36, 44)]. As other emerging biomarkers we mention contents of exosomes (34, 48) [e.g., their miRNAs (49)].

The Cancer Genome Atlas (TCGA) database is a publicly funded project with a primary aim of discovering major genomic changes that cause cancer (50). Use of data from TCGA has assisted in the discovery of new prognostic biomarkers for breast cancer, including (i) miRNAs [miR-574-3p and miR-660-5p (51), and miR-18a, miR-205, and miR-744 (52)], (ii) Piwi-interacting RNAs (piRNAs) and PIWI genes (53), (iii) RNA-protein complexes [tristetrapolin (TTP ZFP36) (54) and Musashi RNA-binding protein 2 (55)], and (iv) gene expressions of mutations [GATA3, NCOR1, CDH1, and ATM (56)].

Thus, in the present paper, we conducted genetic analysis of TCGA database for breast cancer and normal tissues, where it was found that expression of B4GALNT2 gene in breast cancer tissues was higher compared to adjacent normal tissues. This prompted us (i) to examine correlations between knockdown of this gene and the malignancy of breast cancer by carrying out lentivirus-assisted B4GALNT2 gene knockdown experiments in model TNBC cell lines of HCC1937 and MDA-MB-231, and (ii) to investigate the possible mechanisms in play in the downstream pathway of the B4GALNT2 gene by conducting co-immunoprecipitation (Co-IP) mass spectroscopy-assisted analysis and performing *in-vitro* tests. To our knowledge, previous studies pertaining to elucidate correlations between B4GALNT2 gene and cancers were limited to colon cancer (57–59) and lung cancer (60), meaning that the present paper is the first study to address this issue for breast cancer. It should be added that B4GALNT2 gene is a newly discovered antigen in xenotransplantation (61), and its expression changes susceptibility to Salmonella infection (62).

## Materials and Methods

### Analysis of the Differential Expression of B4GALNT2 Gene in Breast Cancer and Non-Tumor Tissues

The RNA-Seq related data of breast cancer tissues and non-tumor tissues were downloaded from the TCGA database. The limma R software package was used for Wilcoxon test to compare the differential expression of B4GALNT2 gene in tumor tissues and non-tumor tissues (|logFC|>2 & adj. P-value<0.01), and drawing the volcano map in R language. Afterwards, the differential expression of the B4GALNT2 gene in the paired cancer and healthy adjacent tissues was compared, and a comparison chart with R language was drawn. For comparing the differential expression of the target gene in the paired cancer and the adjacent healthy tissues, first the dispersion was estimated, and afterwards, general linear model was applied to determine whether there were genetic differences between the different groups. Genes with a statistical test P-value <0.05 were considered differentially expressed genes that meet the null hypothesis. Simultaneously, the differential multiple of gene expression among different groups was calculated. The calculation method used here was Log2 (Cancer/normal), and the filtering standard was ≥1 and ≤-1.

### Cell Lines and Cell Cultures

MDA-MB-231 and SK-BR-3 human breast cancer cell lines (purchased from Shanghai Jikeji Company) were cultured in 37°C constant temperature incubator (containing 5% CO2) [Sanyo Sanyo, Mco-175] with complete medium prepared with DMEM (high glucose) medium (Corning, USA) + 10% fetal bovine serum [FBS (Corning, USA)] + 1% di-anti-penicillin/streptomycin [P/S (Corning, USA)]. HCC1937, T47D and MCF7 human breast cancer cell lines (purchased from Shanghai Jikaine Company) were cultured in a 37°C constant temperature incubator (containing 5% CO2) [Sanyo Sanyo, Mco-175] in a complete medium with RPMI1640 medium (Corning, USA) + 10% fetal bovine serum (FBS) + 1% double-resistant penicillin/streptomycin (P/S).

### Real-Time Quantitative PCR (RT-qPCR)

RNA was isolated using the RNA extraction kit from Tiangen (Haidian, Beijing, China) and the manufacturer’s advised procedure was followed. All the primers were designed and synthesized by Shanghai Jikai Gene Company, and their sequences were listed in **Table S1**. RT-qPCR analysis (with 5×10^5^ cells as samples) was performed using Quant Studio 5 Flex real-time PCR instrument (Thermo Fisher Scientific, Pudong and Shanghai, China). GAPDH was used as the internal reference and B4GALNT2 as the target gene. RNA expression was detected by a two-step RT-qPCR kit (Takaro, Japan), and the relative expression was analyzed by 2-δδCt method. All the experiments were run in triplets.

### Construction of Breast Cancer Cell Lines With Low Expression of B4GALNT2

#### Construction and Packaging of Lentivirus Vectors

The construction and packaging of lentivirus vectors used in this study were all carried out by Shanghai Jikai Gene Company.

#### Lentivirus Transfection of MDA-MB-231 and HCC-1937 Cell Lines

(1) The optimal virus MOI and solution composition were determined through preliminary experiments. The optimal infection efficiency was found to be under the conditions of complete culture medium, 5 μg/mL polybrene, infection enhancer and MOI=20.

(2) After digestion, the cells in logarithmic growth phase were resuspended with complete medium and counted with cell counting plate. The cell concentration was adjusted to 1×10^5^/mL, and two groups were set for each cell line (shCtrl and shB4GALNT2), where two duplicate wells were set in each group. After marking, 1 mL cell suspension and 1 mL complete medium were added to the corresponding wells in the 6-well plate, and the samples were mixed as many times as possible to reduce the error arising from the number of cells between the wells. After adding samples, they were put into a constant temperature incubator at 37°C and 5% CO2 for further culturing overnight.

(3) Preparation before transfection comprised of putting the virus on ice, thawing it and subsequent dilution using serial dilution steps according to the optimal MOI. The growth state and density of cells were observed under a microscope (20% was the best). The old medium was discarded, and complete medium, 5μg/mL polybrene, infection enhancer and virus diluent MOI=20 were added according to the optimal transfection conditions in the preliminary experiment. After mixing, the cells were placed in a constant temperature incubator at 37°C and 5% CO_2_ for further culturing.

(4) After culturing for 12 h, the growth status of cells was observed under a microscope, and the medium was replaced with a fresh one and the cells were put into a constant temperature incubator for further culture. During this period, the growth status of the cells was regularly observed, and the culture medium was replaced with fresh one at appropriate times. After 72h of culture, the 6-well plate was removed from the cell incubator and observed under an inverted fluorescence microscope (100x/200x background; Olympus -IX71, Japan) to observe the expression of green fluorescence.

#### RT-qPCR Detection of Interference Efficiency of Lentivirus Transfected MDA-MB-231 and HCC-1937 Cells

According to the experimental design, each cell was divided into shCtrl and shB4GALNT2 transfection groups, and the relative expression level of gene B4GALNT2 mRNA was detected after MDA-MB-231 and HCC-1937 were transfected by lentivirus. The details of experiments were identical to what is described in subsection 2.3.

### Western Blot Test

First the total cell protein was extracted and its concentration was measured. The gel was prepared according to the instructions of Solarbio’s SDS-PAGE gel preparation kit, and protocols for (i) loading (15 μL per well), (ii) electrophoresis (in the concentrated gel, apply constant voltage modulation 80V, run for 30 minutes; once the marker is separated, adjust the voltage to 120V for 60 minutes), (iii) transferring of the membrane (a sponge pad, 3 layers of filter paper, gel plate, PVDF membrane, 3 layers of filter paper, a sponge pad from the negative plate should be put on the positive plate; the constant current of 300 mA at 4 °C for 150 min should be applied), (iv) blocking (TBST solution containing 5% skim milk), (v) incubation of primary antibody (Anti-B4GALNT2-Sigma-HPA015721; Anti-GAPDH-Santa-Cruz-sc-32233; dilute 1:1000, incubate for 2 h at room temperature) and (vi) incubation of secondary antibody (mouse IgG-CST-#7076; rabbit IgG-CST-#7074; dilute 1:10000, incubate for 2h at room temperature) were followed.

### Celigo Experiments

Two groups of lentivirus transfected cells were prepared and the cell concentration was adjusted to 2000 cells/100 μL. After the 96-well plate was inoculated, the reading plate was detected with Celigo (full field cell analyzer) [Nexcelom] once a day, starting from the next day for 5 consecutive days.

### MTT Experiments

Two groups of lentivirus transfected cells were prepared and the cell concentration was adjusted to 2000 cells/100 μL. Five 96-well plates were laid (tests for 5 days), they were marked, and divided into two groups (blank shCtrl control group and shB4GALNT2 group). Each experimental group was set with 3 multiple wells, and 100 μL cell suspension was added to each well, and the culture continued. One plate was taken out on the first day after plating, and 20 μL 5 mg/mL MTT (Genview, JT343) was added into the well 4 h before the termination of culture without changing the medium. 4 h later, the culture medium was completely sucked off, where care was taken not to suck off the formazan particles at the bottom of the well plate. 100 μL DMSO was added to each well to dissolve the formazan particles. The oscillator was oscillated for 2-5 min, and the value of optical density (OD) was determined by a plate meter (Tecan Infinite, M2009PR) at 490 nm. The test lasted for 5 days.

### Apoptosis Test

The lentivirus-transfected cells of the two groups were prepared and the cell concentration was adjusted to 5×10^5^/mL. Three wells were set in each group, and 1 mL cell suspension and 1 mL complete medium were added to each well in a six-well plate, and the mixture was mixed for further culture for 48 h. All the cells in each well were collected into a tube, centrifuged at 2000 rpm for 5 min. The supernatant was discarded, and the cells were washed and precipitated twice with PBS. The cells were resuspended with 100 μL 1×binding buffer solution in each tube, and 5 μL of Annexin V-APC was added into the corresponding tubes for staining, mixing, and incubation at room temperature in the dark for 15-20 min. 10μL of 7AAD dye was added into corresponding tubes, which were subsequently incubated at room temperature (avoiding the light) for 10 min. 400 μL 1×binding buffer was added into each tube. Afterwards, flow cytometry tests were conducted in triplets.

### Cell Cycle Assay

The lentivirus-transfected cells of the two groups were prepared and the cell concentration was adjusted to 5×10^5^/mL. Three wells were set in each group, and 1 mL cell suspension and 1 mL complete medium were added to each well in a six-well plate, and the mixture was mixed for further culture for 48 h. All the cells in each well were collected into a tube, centrifuged at 2000 rpm for 5 min. The supernatant was discarded, and the cells were washed and precipitated twice with PBS, and each tube was added with 500 μL 70% ethanol precooled at -20°C (70% anhydrous ethanol + 30% PBS) to resuspended the cell precipitation. The cells were placed overnight in a refrigerator at 4°C or placed at -20°C for 2 h to fix the cells. After centrifuging and discarding the supernatant, 500 μL of the prepared PI staining solution was added, and incubation was carried out for 30 minutes at room temperature in the dark. Afterwards, the flow cytometry tests were conducted in triplets.

### Transwell Experiments

The transwell plate can detect the migration and invasion ability of cells. Matrigel needs to be injected into the chamber in advance to detect the invasion ability. Matrigel is not required to detect the migration ability. The rest of steps for these two types of experiments are the same. MDA-MB-231 and HCC1937 cells were re-suspended in serum-free medium (1×10^5^ cells/mL). The suspension (200 μL) was seeded into the upper chamber (the pore size was 8 μm; CoStar, Cambridge, Massachusetts). Afterwards, 600 μL of complete medium containing 10% FBS was added to the lower chamber. After incubation for 28 h, the non-migrated cells on the upper side of the transwell chamber were gently swabbed. Next, the cells fixed on the lower side of the compartment were fixed with 4% paraformaldehyde and stained with 0.1% crystal violet. Finally, after washing three times with PBS and air-drying, the photos were taken using an inverted microscope (Leica MZ8, Leica Microsystems, Wetzlar, Germany). Five random high-magnification views for each chamber were selected. The experiments were conducted in triplets.

### Tumor Formation Experiment in Nude Mice

The experimental animal model was 4-week-old female BALB/c nude mouse (purchased from Beijing Vitong Lihua Experimental Animal Technology Co., Ltd.), where a total of 20 mice were used for experiments with 10 mice in each group. MDA-MB-231 cells transfected with shCtrl or shB4GALNT2 lentivirus were subcutaneously injected into nude mice. A sufficient number of lentivirus-transfected cells were cultured, resuspended in medium without fetal bovine serum (concentration = 1×10^7^ cells/mL), and inserted into an icebox. At the same time, Matrigel was placed in the icebox to slowly dissolve. The Matrigel and the cell suspension in the icebox were blended in a 1:1 ratio, the right armpit of the mouse was sterilized, 400 μL of cell suspension for each subcutaneous injection was taken, marked, and injected. The growth status and tumor data of the mice (including the body weight and tumor weight and volume) were collected during a period of about 1 month. Recording of the data was carried out twice a week for a total of 6 times. After the data recording was completed, the mice were killed by cervical dislocation, the tumor tissue was dissected and weighted, and the mice and the tumor were photographed. Data were statistically analyzed to compare the differences in tumor volume and weight between the two groups.

### Co-Immunoprecipitation

Co-IP mass spectroscopy-assisted method was employed to screen the target gene interaction protein. Thus, MDA-MB-231 cells were infected by constructing an overexpressed lentivirus fused with 3×FLAG tag (3×FLAG-bait) at the N end of the target gene (bait). Overexpressed stable strains and stable strains of the control were screened, cell proteins were collected, Anti-Flag antibody was employed for Co-IP, gel electrophoresis was run for identifying the bands, bands were cut and examined by LC-MS/MS testing and analysis, and method of Bios Protein was employed for further verification of Co-IP for candidate proteins. For screening the positive interaction protein, an interference or overexpression vector was constructed to interfere with the target gene in a cell line. Changes in cell proliferation ability were observed through Celigo instrument for 5 consecutive days to verify the functional role of the protein in the downstream mechanism of the gene.

### Construction of Stable Cell Lines

The cells were cultured in the logarithmic growth phase, and the concentration of puromycin was determined for the target cells (the lowest concentration of the drug to kill all the cells was the best concentration). Afterwards, the target cells were infected with lentivirus for 72 h, and the cell status and infection efficiency were observed. It was required that the cells were in good condition without a large number of deaths, so that to ensure that the cell status of the NC group and the control group are equivalent. An appropriate concentration of antibiotics was added and screening was applied for at least 48 h. If the viral vector had a fluorescent label, after the fluorescence efficiency reached 100%, the antibiotic maintenance concentration was reduced (by 1/2~1/4 or lower compared to the initial previous concentration) to continue the screening and amplification of infected cells, and collecting cells for downstream RT-qPCR detection (to identify the expression level of the target gene).

### In-Gel Hydrolysis

Each sample strip (about 1 mm^3^/piece) was chopped; 1 mL 100 mM NH4HCO3/30% ACN was added to decolorize until sample becomes transparent, the supernatant was removed and freeze-dried; 180 μL 100 mM NH_4_HCO_3_ and 20 μL 100 mM DTT were added, incubation was conducted at 56°C for 30 min, the supernatant was removed, 100 μL CAN was added and it was allowed to stand for 5 minutes. Afterwards, the supernatant was sucked off; 140 μL 100 mM NH_4_HCO_3_ and 60 μL 200 mM IAA were added and reaction was conducted in the dark for 20 minutes. The supernatant was removed, 200 mM NH_4_HCO_3_ was added, and it was left at room temperature for 15 minutes. Next, the supernatant was removed, 100 μL ACN was added and it was left for 5 minutes, afterwards, the supernatant was sucked off and was lyophilized. 20 μL 2.5-10ng/μL trypsin solution was added and placed in a refrigerator at 4°C for 30 min. Next, about 40 μL 25 mM NH4HCO3 solution was added and reaction was conducted at 37°C for 20 h. The enzymatic hydrolysate was sucked out, transferred to a new Eppendorf tube, and 100 μL 60% ACN/0.1% TFA was added to the original tube, followed by ultrasound treatment for 15 min. The solution was sucked out and incorporated into the previous solution. Freeze-drying was applied to the mixture. Next, re-dissolution was carried out with 0.1% TFA, followed by de-saltation and lyophilization. Afterwards, re-constitution was carried out by 20 μL 0.1% FA and Q Exactive mass spectrometry was employed.

### Mass Spectroscopy Analysis

Each sample was separated using the Easy NLC system with a nanoscale flow rate. Buffer solution A was 0.1% formic acid aqueous solution, and buffer solution B was 0.1% formic acid acetonitrile aqueous solution (acetonitrile was 80%). The chromatographic column was balanced with 100% liquid A, and the samples were loaded from the automatic sampler to the analytical column (Thermo Scientific, Acclaim PepMap RSLC 50 μm X 15cm, Nano Viper, P/N164943) at A flow rate of 300 nL/min. The linear gradient of liquid B during the separation process was the following: 0%-6% (during 0 min-5 min), 6%-28% (during 5 min-45 min), 28%-38% (during 45-50 min), and 38%-100% (during 55-60 min).

After chromatographic separation, the samples were analyzed by Q Exactive mass spectrometry. The duration of analysis was 60 min, the detection method was positive ion, the scanning range of precursor ion was 350-1800 m/z, the resolution of primary mass spectrometry was 70,000, the AGC target was 3E6, and the primary maximum IT was 50 ms. The mass-to-charge (m/z) ratios of peptides and peptide fragments were collected according to the following methods: 10 fragment patterns were collected after each full scan (MS2 scan), MS2 activation type was HCD, the isolation window was 2 m/z, the secondary mass spectrometry resolution was 17500, microscan was 1, secondary maximum IT was 45 ms, and normalized collision energy was 27 eV.

### Mass Spectrometry Raw File Processing

This project used the Proteome Discoverer 2.1 (Thermo Fisher Scientific) software to convert the original map files (.raw files) generated by Q ExActive into.mgf files, which were submitted to the Mascot 2.6 server for database retrieval through the built-in tools of the software. Next, through Proteome Discoverer 2.1, the database file (.dat file) formed on the Mascot server was sent back to the software, and the data were screened according to the standard of FDR<0.01 to obtain highly credible qualitative results.

### Statistical Method

IBM SPSS Statistic 23.0 software was used for statistical analysis of the experimental data, and Graph Pad Prism8.0 software was used for making images. Each experiment was repeated three times independently, and the results were expressed as mean ± standard deviation (SD). The T-test was used to compare the differences between the two groups of data. F-test (i.e., homogeneity of variance test) was carried out for the two groups of data to be analyzed. If the F-test value was >0.05, the T-test value was obtained by the isovariance double sample test; if the F-test value was <0.05, the T-test value was obtained by the heteroscedasticity double sample test. P-value was taken on both sides, and P <0.05 was considered statistically significant difference between the two samples, where the following symbols were chosen: *, 0.01≤P <0.05; **, P < 0.01, ***, P < 0.001, ****, P < 0.0001.

## Results

### B4GALNT2 Is Highly Expressed in Breast Cancer Cells

**Figure 1:**
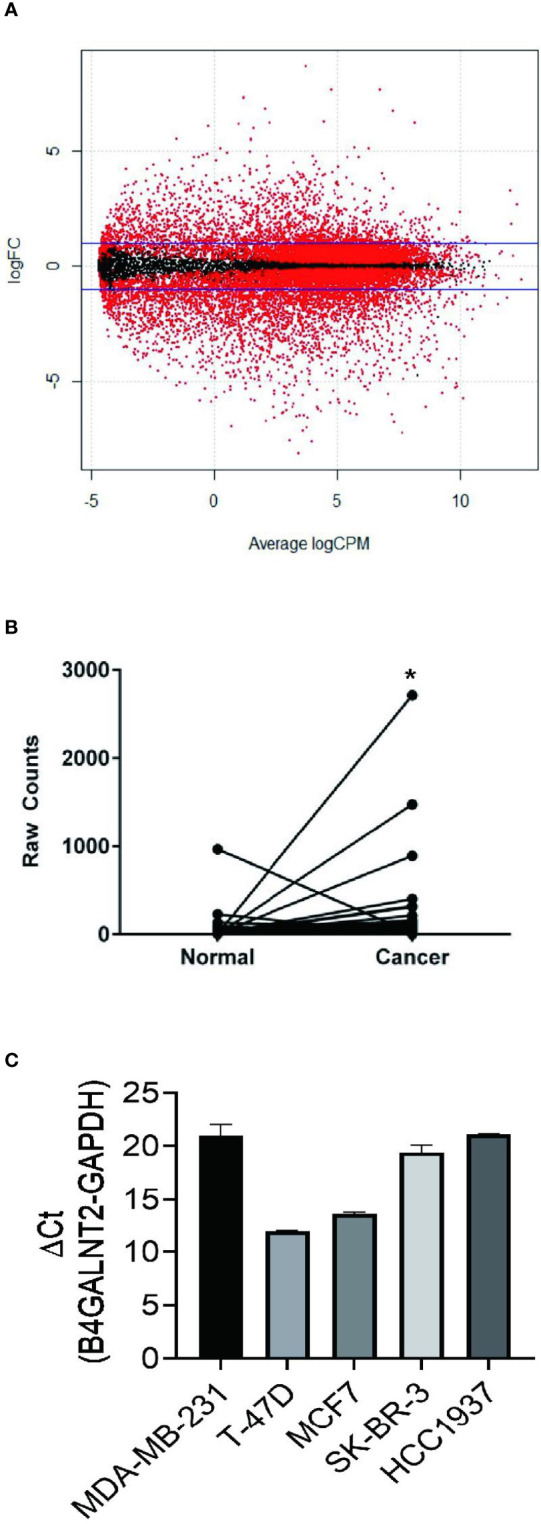
B4GALNT2 gene is highly expressed in breast cancer tissues and cell lines. **(A)** The volcano map of the differential expression of genes between 1094 breast cancer tissues and non-tumor tissues (red corresponds to p<0.05; black corresponds to p>0.05). **(B)** A paired diagram for the expression levels of the B4GALNT2 gene in 106 pairs of breast cancer and normal adjacent tissues. **(C)** The relative expression level of gene B4GALNT2 in five different breast cancer cell lines. *p < 0.05.

Differential expression analysis of RNA-Seq between breast cancer tissue and non-tumor tissue was carried out. RNA-Seq relevant data of breast cancer patients were downloaded from the TCGA database. A comparison was made between 1094 breast cancer and non-tumor tissues to determine the differential expression of B4GALNT2 gene, and the corresponding volcano map was constructed (**Figure 1A**). The differential expression of B4GALNT2 gene in adjacent and cancerous tissues was analyzed. Results of the analysis of differential expression of B4GALNT2 gene in breast cancer and adjacent normal tissues indicated that this gene was highly expressed in the former (**Figure 1B**). Using RT-qPCR, we examined the relative expression level of gene B4GALNT2 mRNA in five different breast cancer cell lines, namely MDA-MB-231 (ER-/PR-/HER2-), T47D (ER+/PR+/HER2-), MCF7 (ER+/PR+/HER2-), SK-BR-3 (ER-/PR-/HER2+) and HCC1937 (ER-/PR-/HER2-) (**Figure 1C**) (63).

### Lentivirus-Assisted Transfection of Cell Lines With Knocked Down B4GALNT2 Gene and Determination of the Knockdown Efficiency

**Figure 2:**
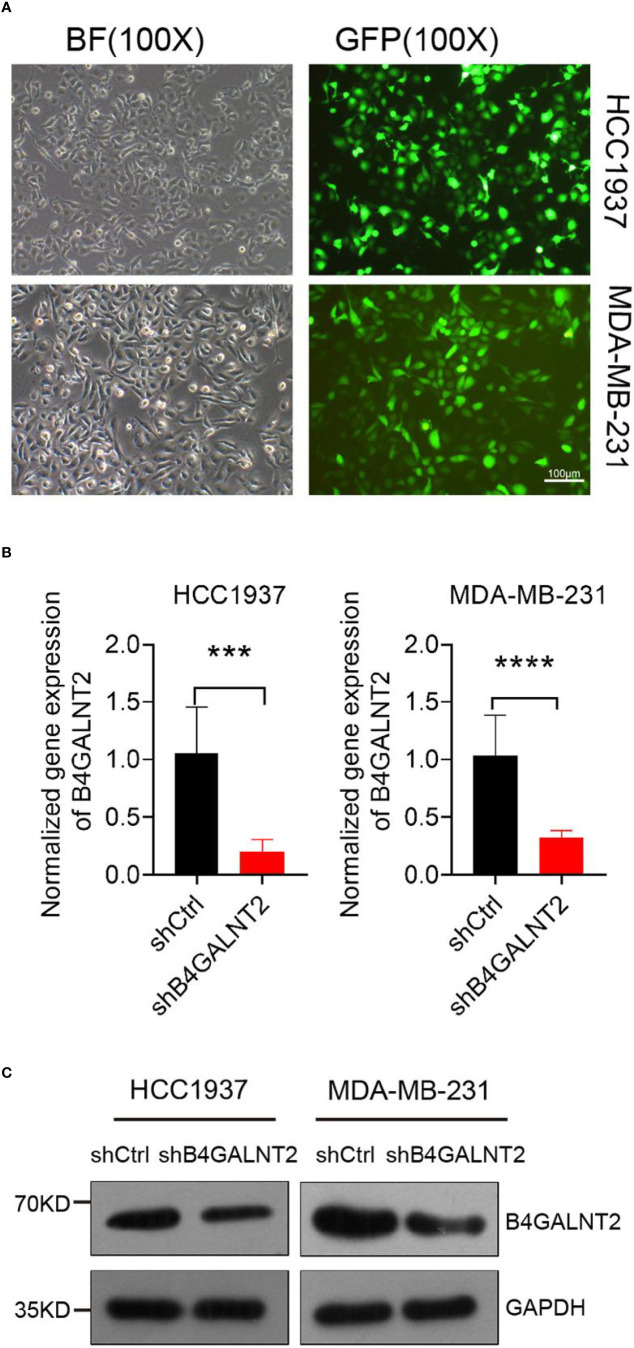
Construction of the cell model. **(A)** Lentiviral transfection efficiency of two breast cancer cell lines. **(B, C)** The mRNA and protein expression levels of B4GALNT2 gene were detected by RT-qPCR and Western blot after 72h of transfection with lentivirus. The success of the model construction is reflected in the joint results of **(B, C)**. ***p < 0.001, ****p < 0.0001.

Lentivirus-assisted transfection of HCC1937 and MDA-MB-231 cell lines with knocked down B4GALNT2 gene (shB4GALNT2) was carried out. Empty lentivirus (shCtrl) transfectant controls were also prepared. Fluorescence microscope images of shB4GALNT2 transfected cell lines taken after 72 hours are shown in **Figure 2A**. The efficiency of B4GALNT2 gene knockdown was evaluated quantitatively through measuring the relative expression level of gene B4GALNT2 mRNA (**Figure 2B**), and qualitatively by carrying out Western blot tests to detect the expressed B4GALNT2 protein (**Figure 2C**). RT-qPCR tests showed that lentivirus transfected cell lines exhibited ~80% (HCC1937) and ~70% (MDA-MB-231) reductions in the relative mRNA levels of the gene (in comparison with shCtrl transfectants) (**Figure 2B**). Also, Western blot tests showed that the level of expression of B4GALNT2 protein decreased upon gene knockdown (**Figure 2C**). Thus, it was confirmed that the lentivirus transfected cell lines possessed low expression levels of B4GALNT2 gene.

### Effects of B4GALNT4 Gene Knockdown on Cell Proliferation, Viability, Apoptosis, Cycles, Migration Ability, and Invasiveness Ability *In Vitro*

**Figure 3:**
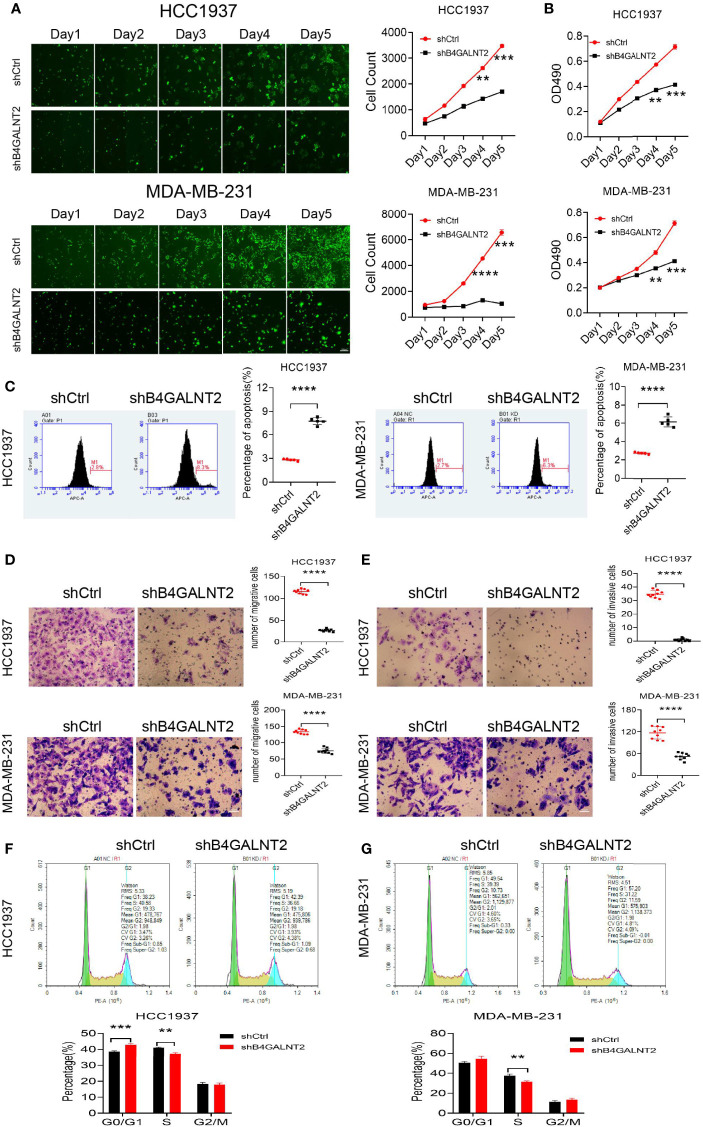
The effects of B4GALNT2 gene on the cell proliferation, invasion, migration and cycle of breast cancer cell lines were investigated by *in-vitro* cell experiments. **(A)** Using Celigo instrument, through counting for 5 consecutive days, it was found that the proliferation ability of the cells of the shB4GALNT2 group were significantly lower than that of the shCtrl group; **(B)** MTT assay was used for detection of the cell viability. After 5 consecutive days of detection, it was observed that the cell viability of shB4GALNT2 group was significantly lower than that of shCtrl group. **(C)** Flow cytometry was used for determining the early apoptosis rate of cells transfected with lentivirus, where it was found that the apoptosis rate of cells in shB4GALNT2 group was higher. **(D, E)** Transwell assay was used for measuring the migration and invasion abilities of cells after knockdown of B4GALNT2 gene, where it was found that the migration and invasion ability of cells in shB4GALNT2 group were significantly reduced. **(F, G)** Flow cytometry was used for detecting the cell cycle, where it was found that the B4GALNT2 gene knockdown resulted in significant G1 phase arrest. **p < 0.01, ***p < 0.001, ****p < 0.0001.

Celigo instrument was used for studying the proliferation of lentivirus (shB4GALNT2 and shCtrl) transfected cell lines. The results are shown in **Figure 3A**. It was found that proliferation of cell lines with knocked down B4GALNT2 gene was inhibited. For example, after 5 days, the inhibition in cell proliferation was ~2.0-fold (for HCC1937 cells) and ~6.4-fold (for MDA-MB-231 cells), in comparison with empty lentivirus (shCtrl) transfectants.

MTT assay was used for studying the cell viability of lentivirus (shB4GALNT2 and shCtrl) transfected cell lines. The results are shown in **Figure 3B**. It was found that cell viability in cell lines with knocked down B4GALNT2 gene was decreased. For example, after 5 days, the reductions in cell viability were manifested in ~1.7-fold (for both HCC1937 and MDA-MB-231 cells) decreases in the absorbance at OD490, in comparison with empty lentivirus (shCtrl) transfectants.

Flow cytometry was used for studying the cell apoptosis of lentivirus (shB4GALNT2 and shCtrl) transfected cell lines. The results are shown in **Figure 3C**. It was found that apoptosis of cell lines with knocked down B4GALNT2 gene was promoted. For example, after 5 days, promotions of cell apoptosis were reflected in ~2.8-fold (for HCC1937 cells) and ~2.1-fold (for MDA-MB-231 cells) increases in the percentage of apoptotic cells, in comparison with empty lentivirus (shCtrl) transfectants.

Flow cytometry was used for studying the cell cycles of lentivirus (shB4GALNT2 and shCtrl) transfected cell lines. The results are shown in **Figures 3F, G**. It was found that in shB4GALNT2 transfected cell lines, the number of cells in the G1 phase increased, in the S phase decreased and did not change in the G2/M phase, in comparison with empty lentivirus (shCtrl) transfectants. The G1 phase exhibited a block.

Experiments were conducted for studying the cell migration ability of lentivirus (shB4GALNT2 and shCtrl) transfected cell lines. The results are shown in **Figure 3D**. It was found that the migration ability of cell lines with knocked down B4GALNT2 gene was inhibited. For example, after 5 days, the inhibition in cell migration ability was ~4.3-fold (for HCC1937 cells) and ~1.8-fold (for MDA-MB-231 cells), in comparison with empty lentivirus (shCtrl) transfectants.

Experiments were conducted for studying the cell invasiveness ability of lentivirus (shB4GALNT2 and shCtrl) transfected cell lines. The results are shown in **Figure 3E**. It was found that the invasiveness ability of cell lines with knocked down B4GALNT2 gene was inhibited. For example, after 5 days, the inhibition in cell invasiveness ability was ~17.0-fold (for HCC1937 cells) and ~2.1-fold (for MDA-MB-231 cells), in comparison with empty lentivirus (shCtrl) transfectants.

### Effects of B4GALNT4 Gene Knockdown on Cell Proliferation *In Vivo*

**Figure 4:**
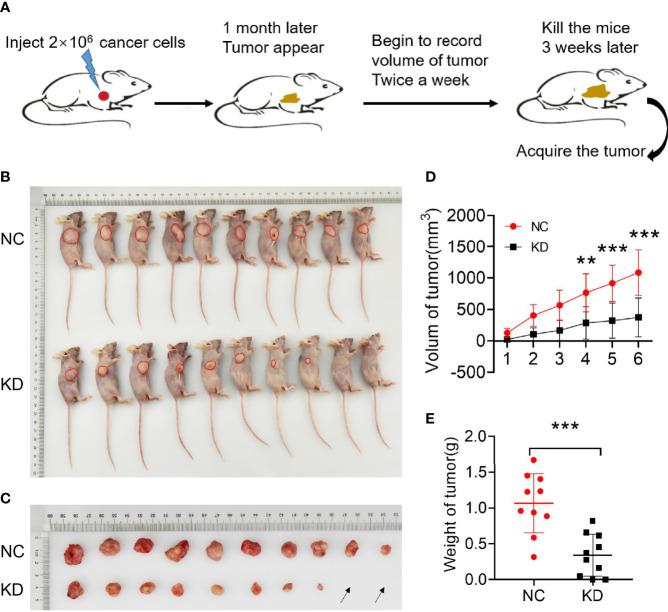
Experiments on animals were conducted to verify the effect of B4GALNT2 gene knockdown of MDA-MB-231 breast cancer cell line on the proliferation of breast cancer cells *in-vivo*. **(A)** Model diagram of the mouse experiments. **(B)** Subcutaneous construction of tumor model in nude mice (each nude mouse was inoculated with 2×10^6^ tumor cells). **(C)** Nude mice tumor size; the black arrow indicates that there was no lump formation. **(D)** Tumor growth curves. **(E)** Statistical comparison of the weight of tumors in the two groups of nude mice. Note: NC=normal control; KD=knockdown of the B4GALNT2 gene. ***p < 0.001, ****p < 0.0001.

*In-vivo* tumor formation experiments in mice were carried out upon success of *in-vitro* experiments, where it was found that knockdown of the B4GALNT2 gene in MDA-MB-231 cells inhibited their proliferation ability (**Figure 4**). For example, for shB4GALNT2 transfected tumors, tumor growth (volume) rate was lower during the one month after tumor construction, and the last tumor volume (**Figure 4D**) was ~2.9-fold smaller (in comparison with shCtrl transfectant tumors). Statistical analysis revealed that the weight of the tumors of the two groups were significantly different (**Figure 4E**).

### Detection of Protein-Protein Interaction Between HLA-B and B4GALNT2 Through Mass Spectrometry-Assisted Co-IP Tests, GO and KEGG Pathway Enrichment Analyses, and *In Vitro* Tests

**Figure 5:**
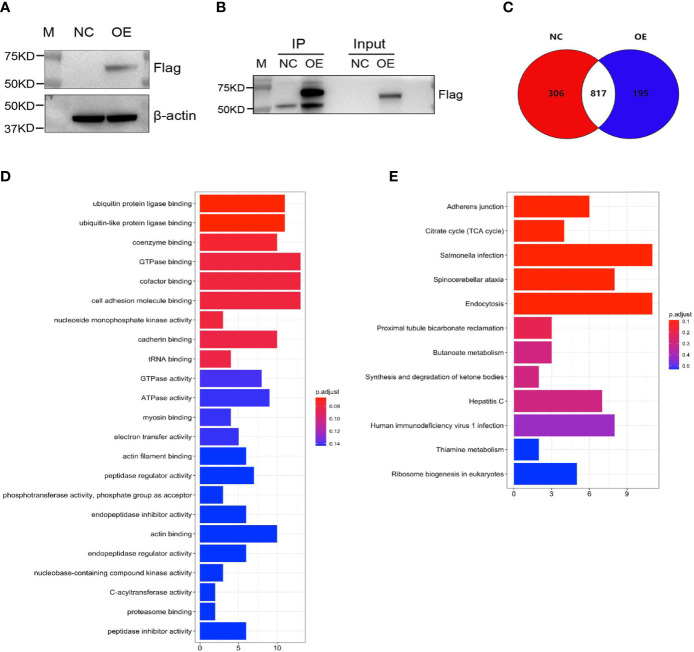
Screening of B4GALNT2 gene interaction proteins by mass spectroscopy-assisted Co-IP. MDA-MB-231 cells were transfected with lentivirus and the target gene was overexpressed. **(A)** Successful overexpression of 3×Flag-fused B4GALNT2 gene. **(B)** Detection of proteins of the whole cell lysate of overexpressed cells (Input-OE) and the normal cell (Input-NC) controls, confirmation of the normal exposure of Flag tag and its recognition by Anti-Flag antibody, and verification of the ability of IP in protein enrichment. **(C)** A Venn diagram illustrates the number of identical and different proteins in the experimental group and the control group. **(D, E)** The results of GO and KEGG pathway enrichment analyses for 195 interacting protein genes identified specifically in the OE group. Note: NC=normal control; OE=overexpression of the B4GALNT2 gene.

**Figure 6:**
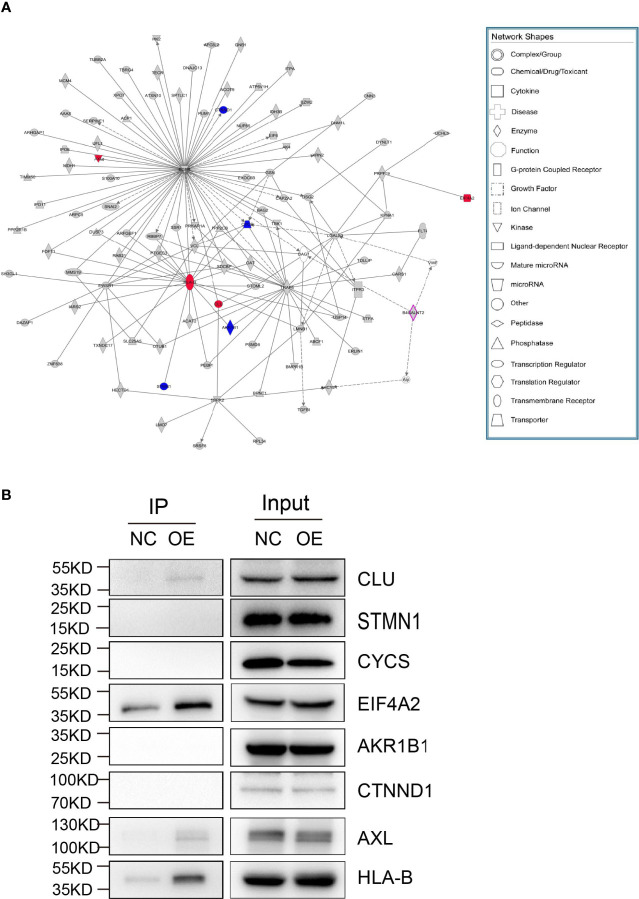
Screening for interacting proteins. **(A)** The genes of the interaction proteins specifically identified in the OE group were identified according to the following conditions: 1) the genes should be predicted to interact with the target gene; 2) the genes should be related to proliferation and metastasis; 3) the genes should be star genes that are reported more frequently in tumors. Such genes were analyzed for gene interaction network, and 8 candidate interacting proteins were finally selected for subsequent Co-IP verification experiments according to relevant literature reports. **(B)** Co-IP validation was performed on 8 candidate interacting proteins. Eight different proteins (CLU, STMN1, CYCS, EIF4A2, AKRIB1, CTNND1, AXL and HLA-B) were checked for their potential interaction with target gene protein, where four of them (CLU, AXL, HLA-B and EIF4A2) exhibited such feature. The gene interaction network diagram shows the interaction relationship network between molecules within a defined functional region. In the network diagram, genes, proteins, chemical substances, and etc. are represented by different shapes. The gray node indicates that the gene belongs to the specific gene in the experimental group, the purple node indicates that the gene is the target gene, and the colorless node indicates that the gene is the added node gene.

**Figure 7:**
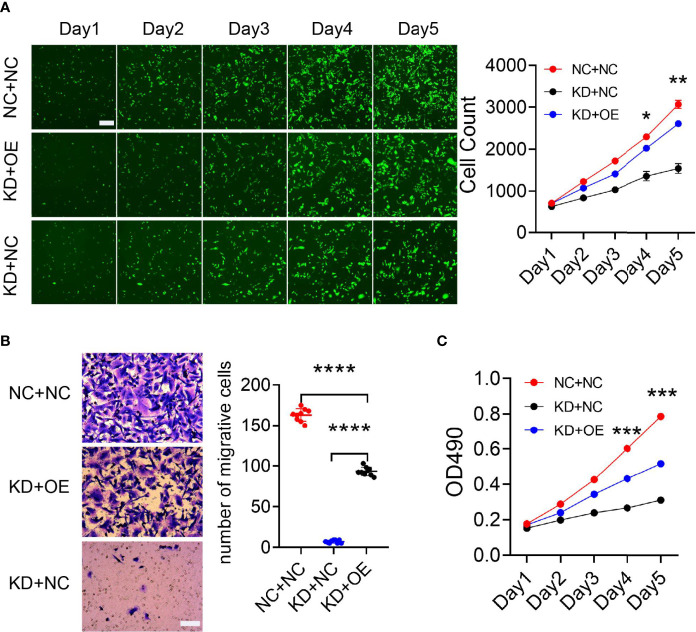
The MDA-MB-231 cell line was used to verify the functional restoration of HLA-B interaction protein genes. **(A)** The proliferation ability of the cells was evaluated with Celigo apparatus for 5 consecutive days. **(B, C)** Transwell plate and MTT reagent were used to detect cell migration and cell viability, respectively. These experiments showed that HLA-B protein could restore the proliferation and migration abilities of breast cancer cells, thus further verifying the existence of an interaction between B4GALNT2 and HLA-B protein molecules. **(A)** Scale bar=100 μm; **(B)** Scale bar=50 μm. Note: NC+NC=normal control group; KD+OE=knockdown of the B4GALNT2 gene + the overexpressed HLA-B gene; KD+NC=knockdown of the B4GALNT2 gene, only. Note: for the 4 positive interaction proteins identified (CLU, AXL, HLA-B and EIF4A2), an interference or expression vector was constructed, thus achieving overexpression of the positive interaction protein while inducing interference with the target gene in a cell line. The Celigo instrument scanned the changes in cell proliferation ability for 5 consecutive days, and verified the functional restoration of the downstream mechanism genes on the target gene. Only the HLA-B gene had an obvious functional restoration effect. *p < 0.05, **p < 0.01, ***p < 0.001, ****p < 0.0001.

Mass spectrometry-assisted Co-IP was applied for elucidating the interaction between HLA-B and B4GALNT2 proteins. For this purpose, first successful overexpression of Flag-fused B4GALNT2 gene was confirmed in the medium by carrying out Anti-Flag antibody pull down experiments in stable cell lines with overexpressed Flag-fused B4GALNT2 gene (OE), as well as for the normal cell (NC) controls (**Figure 5A**). Afterwards, proteins of the whole cell lysate of overexpressed cells (Input-OE) and the normal cell (Input-NC) controls were detected, normal exposure of Flag tag and its recognition by Anti-Flag antibody was confirmed, and the ability of IP in protein enrichment was verified (**Figure 5B**). The number of identical and different proteins in the experimental group and the control group are presented through a Venn diagram (**Figure 5C**). Also, GO and KEGG pathway enrichment analyses were performed for 195 interacting protein genes identified specifically in the OE group (**Figures 5D, E**). Three criteria were applied for determining the interaction proteins specifically identified in the OE group. First, prediction analysis should indicate existence of an interaction between the genes and the target gene. Second, the genes should be associated with proliferation and metastasis. Third, the genes should be among the star genes with high occurrence frequency in tumors. Therefore, a gene interaction network was constructed (**Figure 6A**). Thus, eight candidate interacting proteins (CLU, STMN1, CYCS, EIF4A2, AKRIB1, CTNND1, AXL and HLA-B) were eventually selected for performing Co-IP verification experiments. It was found that four of the candidates interacting proteins (CLU, AXL, HLA-B and EIF4A2) exhibited such feature, and HLA-B was identified to be the one with the highest degree of interaction (**Figure 6B**). For gaining an insight over the interaction between B4GALNT2 and HLA-B proteins, *in-vitro* cell proliferation (**Figure 7A**), cell migration ability (transwell tests) (**Figure 7B**) and cell viability (MTT tests) (**Figure 7C**) experiments were carried out for MDA-MB-231 cell lines. Thus, these experiments were carried out for three types of cell, namely normal control group (i.e., normal target cells plus cells infected with negative control virus; NC+NC), cells with only the knockdown of the B4GALNT2 gene (i.e., cells infected with negative control virus plus cells infected with knocked down B4GALNT2 gene interference; KD+NC), and cells with knockdown of the B4GALNT2 gene plus the overexpressed HLA-B gene (i.e., cells infected with knocked down B4GALNT2 gene interference plus cells infected with overexpression virus of HLA-B; KD+OE). It was found that (i) the proliferation of the KD+NC group was significantly slower than that of the NC+NC group, and (ii) compared with the KD+NC group, the slowing trend of proliferation for KD+OE was significantly restored, indicating that HLA-B protein was an interaction protein for the target gene. For example, after 5 days, the cell proliferation in the KD+NC group was ~50%, and in the KD+OE group was ~85% of that of the NC+NC group, meaning that overexpression of HLA-B restored cell proliferation of the knocked down B4GALNT2 gene by ~35%. Furthermore, MTT and transwell results showed that (i) the cell viability and the cell migration ability for KD+NC group were decreased compared with NC+NC group (P <0.05), and (ii) in KD+OE group these characteristics were increased compared with KD+NC group (P <0.05), indicating that HLA-B gene overexpression could restore the cell viability and metastasis function of the group with knocked down B4GALNT2 target gene, which further corroborates the existence of an interaction between the B4GALNT2 protein and the HLA-B protein. For example, after 5 days, the cell viability and migration ability in the KD+NC group were ~41% and 1%, and in the KD+OE group were ~67% and 57% of those of the NC+NC group, respectively, meaning that overexpression of HLA-B restored cell viability and cell migration ability of the knocked down B4GALNT2 gene by ~26% and 56%, respectively.

## Discussion

Genetic factors play an important role in breast cancer through germline (constitutive) and somatic (acquired) mutations, and so far, many breast cancer susceptibility genes have been identified (64–69). Such genes exhibit variable penetrance, and the well-known ones include high-penetrance genes (BRCA1, BRCA2, TP53, PTEN, STK11 and CDH1) and moderate-penetrance ones (STK11/LKB1, PALB2, CHECK2, ATM, MRE11, RAD50, EAD51, RAD51B, RAD51C, RAD51D, NBS1, BRIP1, FANCA, FANCC, FANCM, and XRCC2), which account for approximately 3-6% and 5-10% of breast cancers, respectively (67).

TNBC constitutes around 12-17% of breast cancers (70), and is notorious for its aggressiveness, which results in late-stage diagnosis (sometimes with metastasis), as well as its difficulty of treatment, due to its ER-, PR- and HR-negative nature that rules out utility of classical receptor-targeting therapies. This means that any progress in identifying and studying susceptibility genes that are associated with TNBC is highly desirable, as it potentially provides carriers of such genes a better screening, risk management and cancer prevention strategies, as well as potentially better therapies for TNBC patient gene carriers through improved prognosis biomarkers and new therapy targets. In this context, we should point out that high-penetrance genes (BARD1, BRCA1, BRCA2, PALB2, and RAD51D) and medium-penetrance ones (BRIP1, RAD51C, and TP53) have been identified for TNBC (71). In fact, up to 15% of TNBC are associated with germline mutations in BRCA1 and BRCA2 (72).

Gene knockdown experiments in model TNBC cell lines are a useful tool to identify the roles played by a gene on processes that involve the cancer cells. Thus, using such experiments it has been found that TNBC cell proliferation inhibition could occur upon knockdown of G-protein–coupled receptor 161 (GPR161) (73), pyruvate kinase muscle (PKM) (74), A-kinase anchor protein 4 (AKAP4) (75), ARHGAP9 (76), and EP300 (77), where promotion/inhibition of other processes has also been identified, e.g., promotion of apoptosis (75, 76) and inhibitions of cell viability (75), cell migration/metastasis ability (75–77), cell invasiveness ability and cell colony forming ability (75, 76). Also, for a model TNBC cell line, knockdown of arylamine N-acetyltransferase I (NAT1) enzyme resulted in a reduced cell invasiveness ability (78).

In the present paper, we investigated the outcomes of the knockdown of B4GALNT2 gene in model TNBC cell lines of HCC1937 (*in-vitro* experiments) and MDA-MB-231 (*in-vitro* and *in-vivo* experiments). B4GALNT2 gene encodes the enzyme β1,4-N-acetylgalactosaminyltransferase 2, which biosynthesizes the histo-blood group antigen Sd^a^ (59). The latter is expressed on the surface of erythrocytes and in body secretions (59). B4GALNT2 gene plays contrasting roles in different cancers. On the one hand, in colon cancer, expression of B4GALNT2 gene was causally associated with a better prognosis (57), where B4GALNT2/Sd^a^ inhibited the stemness-associated malignant phenotype (58). On the other hand, for non-small cell lung cancer (NSCLC) cells, inhibition of B4GALNT2 suppressed proliferation and induced apoptosis in A549 cell lines (60). We should also point out that there is an ongoing clinical trial (phase I/IIa) for B4GALNT2 gene therapy for treatment of duchenne muscular dystrophy (79).

We conducted genetic analysis of TCGA database for patients of breast cancer, where 1094 breast cancer and normal tissues were analyzed for expression of B4GALNT2 gene (**Figure 1A**). It was found that B4GALNT2 gene is overexpressed in breast cancer tumor tissues compared to non-tumor adjacent ones (**Figure 1B**). To further corroborate that B4GALNT2 is highly expressed in breast cancer cells, we examined the relative expression level of gene B4GALNT2 mRNA in five different breast cancer cell lines, where high levels of the corresponding mRNA were found (**Figure 1C**). The two cell lines used for further *in-vitro* knockdown experiments utilizing lentivirus were HCC1937 and MDA-MB-231. Thus, HCC1937 and MDA-MB-231 lentivirus (shB4GALNT2) transfected cell lines and their empty lentivirus (shCtrl) transfectant controls were prepared and examined under the fluorescence microscope (**Figure 2A**). The low expression levels of B4GALNT2 gene in lentivirus transfected cell lines were confirmed quantitatively for its expressed mRNA (see RT-qPCR data of **Figure 2B**) and qualitatively for its expressed protein (see Western blot data of **Figure 2C**). These data indicate that the model construction has been successful.

*In-vitro* experiments were carried out, where it was found that lentivirus (shB4GALNT2) transfection for HCC1937 and MDA-MB-231 cell lines inhibited cell proliferation (**Figure 3A**), decreased cell viability (**Figure 3B**), promoted cell apoptosis (**Figure 3C**), and inhibited cell migration ability (**Figure 3D**) and cell invasiveness ability (**Figure 3E**), in comparison with shCtrl transfectants. In cell cycle tests, it was observed that in shB4GALNT2 transfected cells the G1 phase exhibited a block in comparison with shCtrl transfectants (**Figures 3F, G**). *In-vivo* tumor formation experiments in mice revealed that knockdown of the B4GALNT2 gene in MDA-MB-231 cells inhibited their proliferation ability, in comparison with shCtrl transfectant tumors (**Figure 4**).

**Figure 8:**
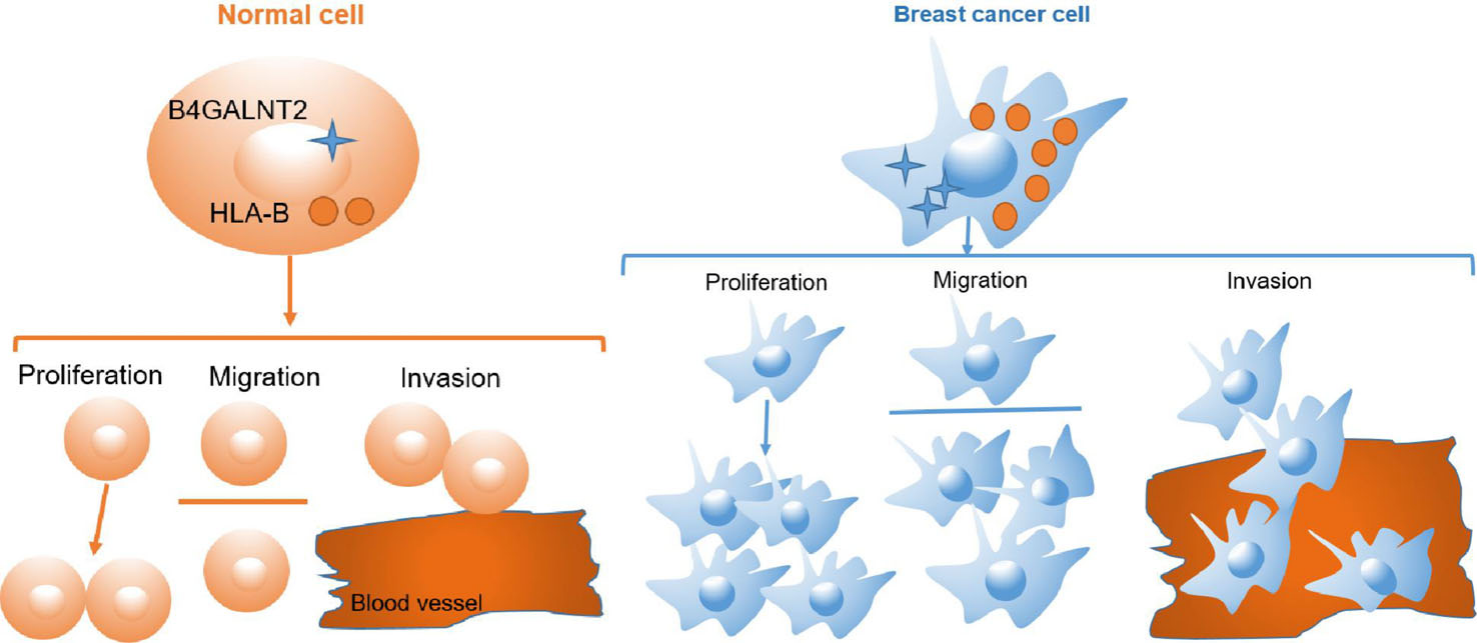
Cell pattern diagram of B4GALNT2 interacting with HLA-B protein to promote breast cancer cell proliferation, migration and invasion.

The human leukocyte antigen (HLA) class I gene products (mainly, HLA-A, -B, and -C) are tasked with presenting endogenous peptides to responding CD8+ T cells (80). In breast cancer, these molecules are downregulated in 40-50% of tumor tissues (81). Their downregulation has been associated with a poor prognosis due to immune-escape mechanism (82, 83). Conversely, expression of HLA-A and HLA-B represents favorable prognosis, because it identifies immune-activated tumors (84). Nevertheless, some reports for breast (85), lung (86) and gastric (87) cancers suggested that high expression of HLA was associated with poor prognosis. In fact, it has been shown that cell-surface stabilization of HLA class I heavy chains by addition of exogenous beta 2-microglobulin (β_2_m) resulted in an increased cell migration ability for medulloblastoma cells via an ERK1/2-mediated mechanism (88, 89). Also, in various model pancreatic cancer cell lines, knockdown of HLA-B increased or decreased migration ability of the cells via, respectively, upregulating or downregulating the expression of integrin beta 1 (ITGB1) [a member of integrin molecules that are implicated in the upregulation of pancreatic cancer cell migration] (90). In this context, we found that there was protein-protein interaction between HLA-B and B4GALNT2 (**Figures 5**, **6**), and overexpression of HLA-B significantly recovered cell proliferation (**Figure 7A**), cell migration ability (**Figure 7B**) and cell viability (**Figure 7C**) of B4GALNT2 gene in MDA-MB-231 cell lines with knocked down B4GALNT2 gene. These indicate that HLA-B is one of the interaction proteins in the downstream pathway of the B4GALNT2 gene (**Figure 8**). Further experiments are needed for gaining a deeper insight over the mechanism of interaction between HLA-B protein and B4GALNT2 gene.

Thus, from the obtained *in-vitro* and *in-vivo* results we may conclude that the status of expression of B4GALNT2 gene in TNBC tissues can potentially be used as a prognosis factor for TNBC patients, and could potentially provide a new therapeutic target. We should point out that given the certain limitations of our study (e.g., the small sample size and no gene restoration or overexpression experiments) the idea of using B4GALNT2 gene as a prognosis factor requires verification through genetic analysis of clinical data.

## Conclusion

Compared with adjacent healthy tissues, B4GALNT2 gene is highly expressed in breast cancer tissues. Knockdown of this gene was negatively correlated with the malignancy of breast cancer. B4GALNT2 may promote the proliferation and metastasis of breast cancer cells by interacting with HLA-B protein. Thus, the B4GALNT2 gene can be used as an indicator to judge the prognosis of breast cancer patients and has the potential as a new therapeutic target.

## Data Availability Statement

The original contributions presented in the study are included in the article. Further inquiries can be directed to the corresponding author.

## Ethics Statement

The animal study was reviewed and approved by Animal Ethics Committee of the First Affiliated Hospital of Zhengzhou University.

## Author Contributions

PY and LZ designed and conducted the experiments, analyzed and interpreted the data, and wrote the manuscript. KC performed the cell-based experiments. YD performed some of the *in-vivo* experiments and advised on the manuscript. CZ performed the biological analysis of the data. WM analyzed patient samples and revised the manuscript. JG conceived the project, supervised the experimental design and data interpretation and wrote the manuscript. All authors contributed to the article and approved the submitted version.

## Funding

This work was supported by the Natural Science Foundation of China (grant number 82070745 to JG), and the Science and Technology Research Project of Henan Province (grant numbers 202102310051 and 182102310571 to JG).

## Conflict of Interest

The authors declare that the research was conducted in the absence of any commercial or financial relationships that could be construed as a potential conflict of interest.

## Publisher’s Note

All claims expressed in this article are solely those of the authors and do not necessarily represent those of their affiliated organizations, or those of the publisher, the editors and the reviewers. Any product that may be evaluated in this article, or claim that may be made by its manufacturer, is not guaranteed or endorsed by the publisher.
