# Supplementary material for: B4GALNT2 Gene Promotes Proliferation, and Invasiveness and Migration Abilities of Model Triple Negative Breast Cancer (TNBC) Cells by Interacting With HLA-B Protein
Source: Front Oncol. 2021 Sep 13;11:722828. doi: 10.3389/fonc.2021.722828 (PMC8473878; doi:10.3389/fonc.2021.722828)
Supplement: Supplementary file 1 [file Table_1.docx]

**Supplementary Information**

Table S1. The sequence of the primers of the reference gene and the target gene are as follows

| Name of the gene | Primer sequence |
| --- | --- |
| GADPH | F: 5’- TGACTTCAACAGCGACACCCA-3’ |
|  | R: 5’- CACCCTGTTGCTGTAGCCAAA-3’ |
|  |  |
| Sh-B4GALNT2 | F:5’- TTTTCCTACGATGGAATCTGGC-3’ |
|  | R:5’- CAGCCTGTCTCCTCGCTTTC-3’ |

The interference sequence of the target gene is as follows:

sh-B4GALNT2：5’-GGTCATAATCCTGGTACTTGG-3’

sh-NC sense : 5’- TTCTCCGAACGTGTCACGT -3’

sh-NC antisense: 5’- ACGTGACACGTTCGGAGAA -3’

The overexpress sequence of the B4GALNT2 is as follows ：

B4GALNT2(46376-1)-P1：CAGCTAGCGTTATTGAATTCATGGGGAGCGCTGGCTTTTCCG

B4GALNT2(46376-1)-P2：TTCCACAGGAATCAGAATTCTTATGCGGCACATTGGAGATGGTTC

The overexpress sequence of the HLA-B is as follows ：

HLA-B(46681-1)-P1：AGGTCGACTCTAGAGGATCCCGCCACCATGCTGGTCATGGCGCCCCGAACC

HLA-B(46681-1)-P2：CACACATTCCACAGGCTAGCTCAAGCTGTGAGAGACACATCAGAGC
